# Mild Cognitive Impairment Is Associated with Enhanced Activation of Th17 Lymphocytes in Non-Alcoholic Fatty Liver Disease

**DOI:** 10.3390/ijms241210407

**Published:** 2023-06-20

**Authors:** Alessandra Fiorillo, Juan-José Gallego, Franc Casanova-Ferrer, Carla Giménez-Garzó, Amparo Urios, Maria-Pilar Ballester, Lucia Durbán, Maria-Pilar Rios, Javier Megías, Teresa San Miguel, Elena Kosenko, Desamparados Escudero-García, Salvador Benlloch, Vicente Felipo, Carmina Montoliu

**Affiliations:** 1 Fundación de Investigación Hospital Clínico Universitario de Valencia-INCLIVA, 46010 Valencia, Spain; alessa.fiorillo@gmail.com (A.F.); juanjo26.gr@gmail.com (J.-J.G.); franc@alumni.uv.es (F.C.-F.); amparo.urios@uv.es (A.U.); 2 Laboratory of Neurobiology, Centro Investigación Príncipe Felipe, 46012 Valencia, Spain; cgimenez@cipf.es (C.G.-G.); vfelipo@cipf.es (V.F.); 3 Servicio de Medicina Digestiva, Hospital Clínico Universitario de Valencia, 46010 Valencia, Spain; mapibafe@gmail.com (M.-P.B.); m.desamparados.escudero@uv.es (D.E.-G.); 4 Servicio de Medicina Digestiva, Hospital Arnau de Vilanova, 46015 Valencia, Spain; durban_luc@gva.es (L.D.); mriosp73@hotmail.com (M.-P.R.); drbenlloch@yahoo.es (S.B.); 5 Departamento de Patología, Facultad de Medicina, Universidad de Valencia, 46010 Valencia, Spain; javier.megias@uv.es (J.M.); teresa.miguel@uv.es (T.S.M.); 6 Institute of Theoretical and Experimental Biophysics, Russian Academy of Sciences, 142290 Pushchino, Russia; eakos@rambler.ru; 7 Departamento de Medicina, Facultad de Medicina, Universidad de Valencia, 46010 Valencia, Spain; 8 CIBERehd, Instituto de Salud Carlos III, 28029 Madrid, Spain

**Keywords:** inflammation, mild cognitive impairment, non-alcoholic fatty liver disease, immunophenotype, interleukins, lymphocyte activation

## Abstract

Patients with nonalcoholic fatty liver disease (NAFLD) may show mild cognitive impairment (MCI). The mechanisms involved remain unclear. The plasma concentrations of several cytokines and chemokines were measured in 71 NAFLD patients (20 with and 51 without MCI) and 61 controls. Characterization and activation of leukocyte populations and CD4^+^ sub-populations were carried out and analyzed by flow cytometry. We analyzed the cytokines released from CD4^+^ cell cultures and the mRNA expression of transcription factors and receptors in peripheral blood mononuclear cells. The appearance of MCI in NAFLD patients was associated with increased activation of CD4^+^ T lymphocytes, mainly of the Th17 subtype, increased plasma levels of pro-inflammatory and anti-inflammatory cytokines such as IL-17A, IL-23, IL-21, IL-22, IL-6, INF-γ, and IL-13, and higher expression of the CCR2 receptor. Constitutive expression of IL-17 was found in cultures of CD4^+^ cells from MCI patients, reflecting Th17 activation. High IL-13 plasma levels were predictive of MCI and could reflect a compensatory anti-inflammatory response to the increased expression of pro-inflammatory cytokines. This study identified some specific alterations of the immune system associated with the appearance of neurological alterations in MCI patients with NAFLD that could be the basis to improve and restore cognitive functions and quality of life in these patients.

## 1. Introduction

Non-alcoholic fatty liver disease (NAFLD) is a progressive disease, ranging from non-alcoholic fatty liver (NAFL) to non-alcoholic steatohepatitis (NASH), where inflammation may be associated with fibrosis, and which may progress to liver cirrhosis and hepatocellular carcinoma [1]. NAFLD is considered the hepatic manifestation of metabolic syndrome and is therefore associated with features of this syndrome such as obesity, type II diabetes, hyperdyslipidemia, and insulin resistance [2]. Given the current epidemic of obesity and metabolic syndrome, NAFLD has become a major cause of chronic liver disease worldwide, with a global prevalence of 25% [3]. 

Different studies have suggested the involvement of metabolic syndrome and its components, including liver manifestations such as NAFLD, in cognitive impairment, ranging from mild cognitive changes to dementia [4,5]. 

In a previous study, we reported that a substantial percentage of patients with NAFLD (32%) show mild cognitive impairment (MCI), characterized by the impairment of selective, sustained, and verbal attention, mental concentration, psychomotor speed, cognitive flexibility, inhibitory mental control, and working memory, with an associated negative impact on everyday living and quality of life [6].

Chronic systemic inflammation is known to be a damaging process and a risk factor for several neurodegenerative and cardiovascular diseases. Chronic inflammatory diseases, such as diabetes, rheumatoid arthritis, and liver cirrhosis, can lead to neurological alterations [7,8,9,10]. In cirrhotic patients with MHE, the reversal of peripheral inflammation by treatment with rifaximin restores the cognitive function [11]. The dysregulation of the peripheral and central immune systems is a predominant feature of neurodegenerative diseases, in which T cell subtypes play an important role in inducing cognitive impairment [12,13]. In a previous study in cirrhotic patients, we demonstrated that mild cognitive alterations (minimal hepatic encephalopathy) were associated with specific changes in peripheral inflammation and immunophenotype, particularly with the activation of Th22, Tfh, CD4^+^CD28^−^, and B lymphocytes. These changes in lymphocyte populations were reflected specifically in the pattern of interleukins they released [14]. Increased levels of certain ILs activate their receptors on endothelial cells, which transmit these alterations to the brain, leading to cognitive and motor impairment [15,16].

In NAFLD, lipid accumulation and hepatocyte injury induce the recruitment of macrophages, i.e., Kupffer cells and monocyte-derived macrophages, that secrete proinflammatory cytokines and chemokines, which facilitates NASH progression [17,18]. 

Multiple T cell subsets are involved in NAFLD pathogenesis, exerting differential effects on adiposity, insulin resistance, steatosis, hepatic inflammation, hepatic injury, and fibrosis [19,20]. Hyperammonemia plays a synergistic role with inflammation in inducing neurologic impairment in liver cirrhosis patients [21,22]. In our previous studies, patients with non-alcoholic steatohepatitis (NASH) without liver cirrhosis showed increased levels of inflammatory factors [23,24] and developed cognitive impairment if the ammonia and inflammation levels were high enough [22]. In rats with diet-induced NASH, neurobehavioral disorders were associated with systemic hyperammonemia, gut dysbiosis, and a deficit of neurotransmitters in several brain regions [25]. Moreover, NAFLD is associated with a smaller total brain volume, which points to a possible link between hepatic steatosis and brain aging [26].

We previously showed the presence of neuroinflammation in post-mortem cerebellums of NASH patients, with an increased activation of microglia and astrocytes, loss of granular and Purkinje neurons, and CD4^+^ T cell infiltration [27,28].

Recently, it was shown that NAFLD accelerated pathological Alzheimer’s disease signs such as neuronal apoptosis and reduced the expression of low-density lipoprotein receptor-related protein-1, involved in β-amyloid clearance [29].

Nonetheless, how inflammation induces cognitive alterations in NAFLD patients is unclear and poorly studied. We hypothesized that, as in cirrhotic patients, MCI appearance in NAFLD patients could be associated with specific qualitative changes in peripheral inflammation and in the immune system that could trigger the induction of cognitive and motor impairment. 

The aim of our study was to characterize these changes in the immunophenotype and peripheral inflammation in NAFLD patients with or without MCI compared to controls without liver disease. A secondary aim was to assess whether these changes were associated with liver injury severity.

## 2. Results

### 2.1. Patient Characteristics

The patient characteristics are shown in Table 1. Overall, 28% of the patients (20 of 71) were classified as with MCI according to the mean ± 2SD criterion [6], showing a significantly lower score (−7.2 ± 0.5) than that obtained by NMCI patients (−1.0 ± 0.3; *p* < 0.0001) or controls (−0.8 ± 0.3; *p* < 0.0001) (Table 1). The patients with MCI performed worse than the NMCI patients in all evaluated psychometric tests (Table S2).

The two patient groups (NMCI and MCI) and the controls had similar demographic (age and sex), educational, and clinical (comorbidities, renal and hepatic function) characteristics. Compared with the control group, the NAFLD patients showed increased levels of liver damage markers such as AST and ALT (Table 1). The blood ammonia levels, although within the normal range, were higher in patients both with (18 ± 3; *p* < 0.001) and without MCI (15 ± 1; *p* < 0.01) than in controls.

According to the NAS and FAST scores, 55% of the patients were classified as NAFL (n = 39), and 45% as NASH (n = 32), and these were homogeneously distributed across the MCI and NMCI groups (Table 1).

The NASH patients showed significantly higher liver fibrosis than the NAFL patients (*p* = 0.023), as shown in Table S3.

### 2.2. Monocyte Populations in Peripheral Blood 

The NAFLD patients showed an increased percentage of intermediate CD14^+^CD16^+^, pro-inflammatory monocytes compared to the controls (*p* < 0.0001) (Figure S1). This increase was associated with a reduced percentage of classical, non-inflammatory CD14^++^CD16^−^ monocytes, which represented 95.2 ± 0.3% of the total population in controls, 93.5 ± 0.5% in patients without MCI, and 92.5 ± 0.7% (*p* < 0.05) in patients with MCI. Furthermore, the NAFLD patients showed a significant decrease in the percentage of non-classical CD14^+^CD16^++^ monocytes, with 0.1 ± 0.0% of the total population (*p* < 0.0001) in patients without MCI and 0.2 ± 0.1% (*p* < 0.0001) in patients with MCI, compared to the control group (0.7 ± 0.1%) (Figure S1).

### 2.3. MCI Association with the Induction of the Early Activation Marker CD69 in CD4^+^ T Lymphocytes in Peripheral Blood

**Figure 1:**
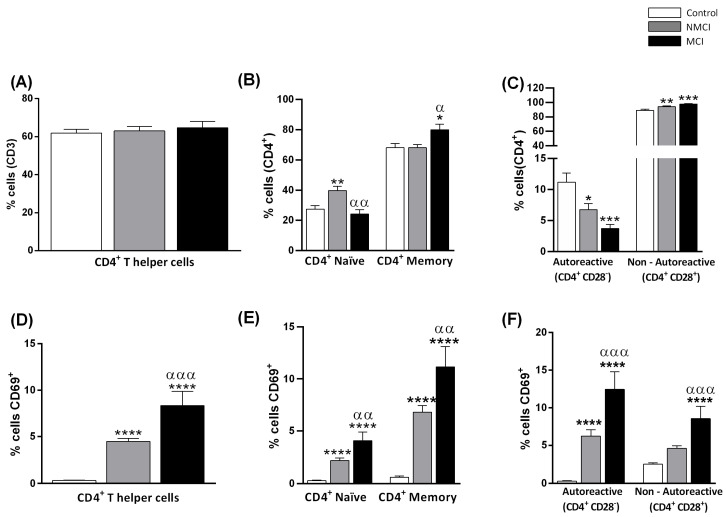
MCI in NAFLD patients is associated with the induction of the early activation marker CD69 in CD4^+^ T lymphocytes in peripheral blood. (**A**) Percentage of CD4^+^ T lymphocytes relative to total lymphocytes. (**B**) Percentage of CD4^+^ T lymphocytes that were naïve or memory. (**C**) Percentage of CD4^+^ T lymphocytes that were autoreactive (CD4^+^CD28^−^) or non-autoreactive (CD4^+^CD28^+^). (**D**–**F**) Percentage of CD69 expression in total CD4^+^ T lymphocytes (**D**), naïve, and memory lymphocytes and (**E**) autoreactive and non-autoreactive lymphocytes (**F**). Values are mean ± SEM of the following groups: controls, n = 29; patients without MCI (NMCI), n = 32; patients with MCI (MCI), n = 14. Values significantly different from the controls are indicated by asterisks (*). Values significantly different in patients with MCI compared to NMCI patients are indicated by (^α^): */^α^ *p* < 0.05; **/^αα^ *p* < 0.01; ***/^ααα^ *p* < 0.001; **** *p* < 0.0001. MCI, mild cognitive impairment.

Although the proportion of CD4^+^ lymphocytes to total (CD3^+^) T lymphocytes was not affected in patients without or with MCI (Figure 1A), the early activation marker CD69 showed increased expression in CD4^+^ T lymphocytes in both NMCI and MCI patients compared with the control group (*p* < 0.0001), with higher activation in MCI than in NMCI patients (*p* < 0.001) (Figure 1D).

The patients with MCI showed a significantly higher percentage of memory CD4^+^ T lymphocytes (80 ± 4%) than the control subjects (68 ± 3%, *p* < 0.05) or the patients without MCI (68 ± 2%, *p* < 0.05) (Figure 1B). The percentage of naïve CD4^+^ T lymphocytes tended to be reduced in parallel in the MCI patients, indicating a shift from naïve to memory cells (Figure 1B).

Both naïve and memory populations of CD4^+^ cells expressed higher levels of the CD69 activation marker in the NAFLD patients than in the controls (*p* < 0.0001). Furthermore, the MCI patients showed greater activation than the NMCI patients, of both naïve (*p* < 0.01) and memory cells (*p* < 0.01) (Figure 1E).

Most CD4^+^ lymphocytes are also CD28^+^ and require exposure to CD28 to be activated. Some CD4^+^ lymphocytes lack CD28 (CD4^+^CD28^−^) and are considered autoreactive. The proportion of CD4^+^CD28^−^ lymphocytes was lower in both MCI (4 ± 1%) and NMCI patients (7 ± 1%) compared with the controls (12 ± 1%; *p* < 0.01 and *p* < 0.05, respectively) (Figure 1C). The percentage of non-autoreactive (CD4^+^CD28^+^) T lymphocytes increased in parallel in patients without and with MCI compared to the controls (*p* < 0.01 and *p* < 0.001, respectively). The MCI and NMCI patients, compared to the controls, exhibited a significant rise in CD69 expression in autoreactive cells (*p* < 0.0001), but only the MCI patients showed CD69 increase in non-autoreactive cells compared with the controls (*p* < 0.0001) (Figure 1F). Moreover, both autoreactive and non-autoreactive cells were more activated in MCI than in NMCI patients (*p* < 0.001) (Figure 1F).

In summary, our results indicated an activation of CD4^+^ T lymphocytes in the MCI patients (Figure 1). To assess whether these changes were associated with liver injury severity, we analyzed the data by the different liver damage degree (NAFL and NASH) (Table S4), finding a significant general activation in all lymphocyte populations tested, irrespective of liver damage.

**Figure 2:**
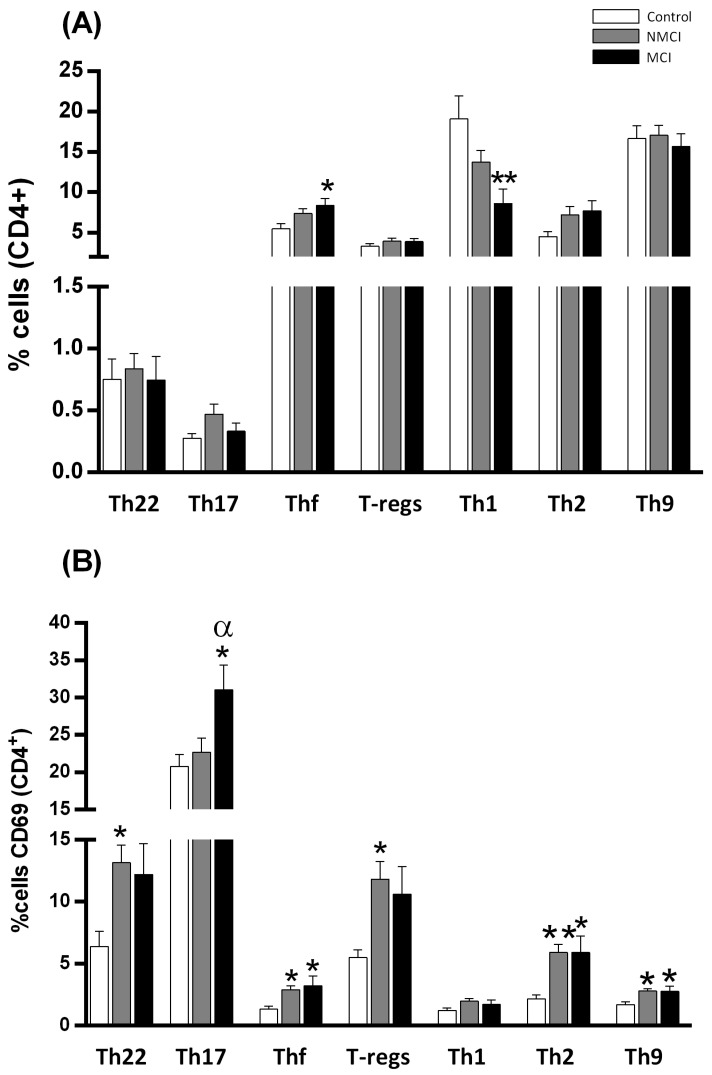
MCI in NAFLD patients is associated with the induction of the early activation marker CD69 in Th17 cells. (**A**) Percentages of subpopulations of T-helper CD4^+^ lymphocytes: Th22, Th17, Thf, T-regs, Th1, Th2. (**B**) Percentages of cells within the subpopulations of T-helper CD4^+^ lymphocytes that expressed CD69. Values are the mean ± SEM of the following groups: controls n = 14; patients without MCI (NMCI) n = 26; patients with MCI (MCI) n = 12. Values significantly different from the controls are indicated by asterisks (*). Values significantly different in patients with MCI compared to NMCI patients are indicated by (^α^): */^α^ *p* < 0.05; ** *p* < 0.01.

Regarding the study of T-helper lymphocyte subsets, the patients with MCI showed significantly heightened Thf differentiation and a lower percentage of Th1 cells compared to the controls (*p* < 0.05 and *p* < 0.01, respectively) (Figure 2A). Although there were no differences in Th17 cell percentages, CD69 expression was increased in Th17 cells of patients with MCI compared with those of patients with NMCI and of the control groups (*p* < 0.05). Overall, the NAFLD patients showed a higher percentage of CD69 cells than the controls, and we found significant differences in Th22, Thf, Th2, Tregs, and Th9 activation both in patients with and without MCI (Figure 2B). It is noteworthy that the activation of Th17 increased selectively in patients with MCI, but not in those without MCI, suggesting an association between the activation of Th17 lymphocytes and the appearance of MCI.

### 2.4. Expression Analysis of Transcription Factors Characteristic of Different CD4^+^ T-Lymphocyte Subsets

**Figure 3:**
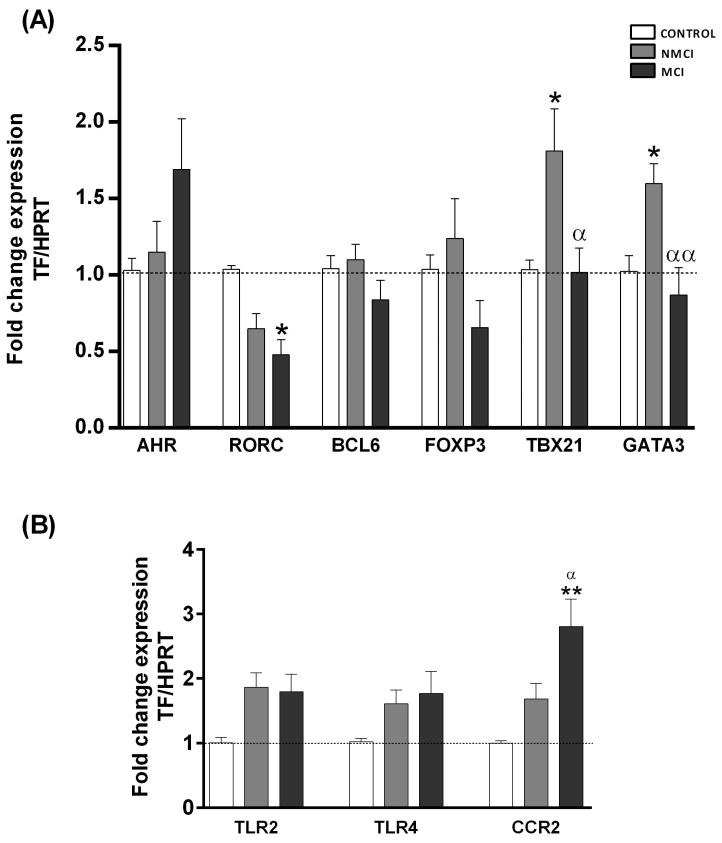
Analysis of the mRNA expression of transcription factors characteristic of different subsets of CD4^+^ T lymphocytes and receptors in peripheral blood mononuclear cells. (**A**) Expression analysis of the transcription factors AHR, RORC, BCL6, FOXP3, TBX21, and GATA3, characteristic of Th22, Th17, Thf, Treg, Th1, and Th2 lymphocytes, respectively. (**B**) Expression analysis of TLR2, TLR4, and CCR2 receptors. Data represent the normalized target gene amount relative to controls, which were considered as 1. Values are the mean ± SEM of the following groups: controls n = 17; patients without MCI (NMCI) n = 16; patients with MCI (MCI) n = 17. Values significantly different from the controls are indicated by asterisks (*). Values significantly different in patients with MCI compared to NMCI patients are indicated by (^α^): */^α^ *p* < 0.05; **/^αα^ *p* < 0.01. MCI, mild cognitive impairment.

CD4^+^ T lymphocytes may differentiate into different subsets, characterized by their expression of specific transcription factors and by the production of certain cytokines. The transcription factor AHR, specific for Th22 lymphocytes, showed a non-significant trend of increased expression in MCI patients compared to the control and NMCI groups, (*p* = 0.112 and *p* = 0.150, respectively), while the transcription factor RORC, characteristic of Th17 cells, was significantly decreased compared to the controls (*p* < 0.05) (Figure 3A). TBX21 and GATA3, specific for Th1 and Th2 lymphocytes, respectively, showed a significantly higher expression in NMCI patients than in the controls (*p* < 0.05), whereas the expression of these transcription factors was significantly decreased (*p* < 0.05, *p* < 0.01 respectively) in the MCI patients compared to patients without MCI. No differences were found for FOXP3 and BCL6, markers of Tregs and Tfh (Figure 3A). 

The expression of the chemokine receptor CCR2 was higher in PBMCs from the MCI patients than in those from the NMCI patients (*p* < 0.05) or the controls (*p* < 0.01). No significant differences were found for TLR4 receptors, whereas in MCI patients, TLR2 was significantly increased compared to the controls (*p* < 0.05) (Figure 3B).

### 2.5. Plasma Levels of Different Cytokines and Chemokines

**Figure 4:**
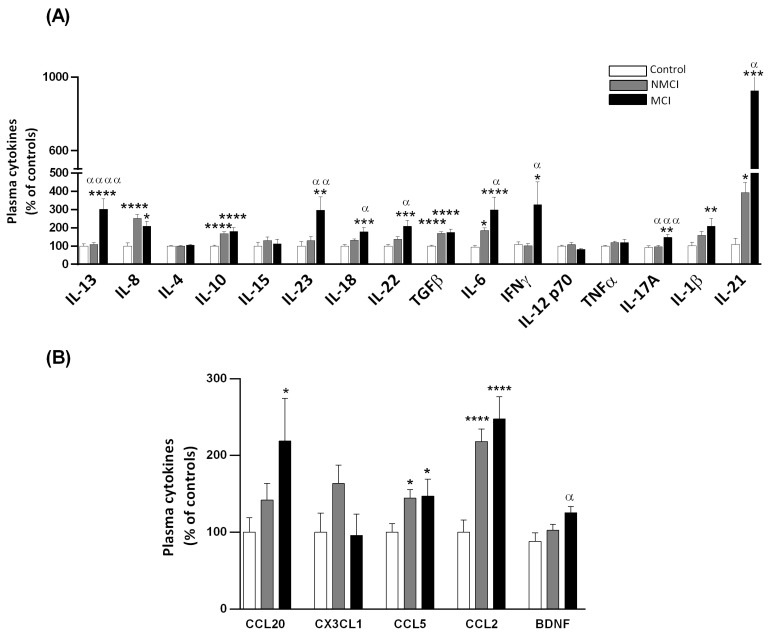
Plasma levels of different pro-inflammatory and anti-inflammatory cytokines and chemokines in NAFLD patients and controls. (**A**,**B**) The plasma levels of cytokines are expressed as percentage of the control levels to allow the easy identification of alterations in patients without MCI (NMCI) and with MCI (MCI). Values are the mean ± SEM of the following groups: control, n = 30; NMCI, n = 49; MCI, n = 22. Values significantly different from the controls are indicated by asterisks (*). Values significantly different in patients with MCI compared to NMCI patients are indicated by (^α^): */^α^ *p* < 0.05; **/^αα^ *p* < 0.01; ***/^ααα^ *p* < 0.001; ****/^αααα^ *p* < 0.0001. MCI, mild cognitive impairment; BDNF, brain-derived neurotrophic factor.

We analyzed a wide range of different pro-inflammatory and anti-inflammatory cytokines and chemokines in the plasma from NAFLD patients and controls (Figure 4). Increases in IL-13, IL-23, IL-18, IL-22, IL-6, IL-17A, IFN-γ, and IL-21 were significantly greater in patients with MCI than in those without MCI or in the controls (Figure 4A). BDNF was also increased in MCI compared to NMCI patients (Figure 4B). In addition, the NAFLD patients with and without MCI showed significantly increased IL-8, IL-10, TGFβ, IL-6, IL-21, CCL5, and CCL2 compared with the control group, whereas the plasma concentrations of IL-1β and CCL20 were significantly heightened only in patients with MCI. No between-group differences were found in the plasma levels of IL-4, IL-15, IL-12 p-70, TNF-α, or CX3CL1 (Figure 4A,B). The plasma concentration of each cytokine in the controls, and in the NMCI and MCI patients are shown in Table S5.

To assess whether these changes were associated with liver injury severity, we grouped the patients according to liver damage (NAFL and NASH) for data analysis (Table S6). The NASH patients showed higher TNF-α levels than the NAFL patients (*p* < 0.01) or the controls (*p* < 0.05) and lower BDNF plasma levels than the NAFL patients (*p* < 0.05). the plasma levels of the cytokines IL-6, IL-10, TGF-β, IL-8, IL-21, and CCL2 were significantly increased in both patient groups compared to the controls (Table S6).

### 2.6. Analysis of Cytokines Released by CD4^+^ T Cell Cultures

**Figure 5:**
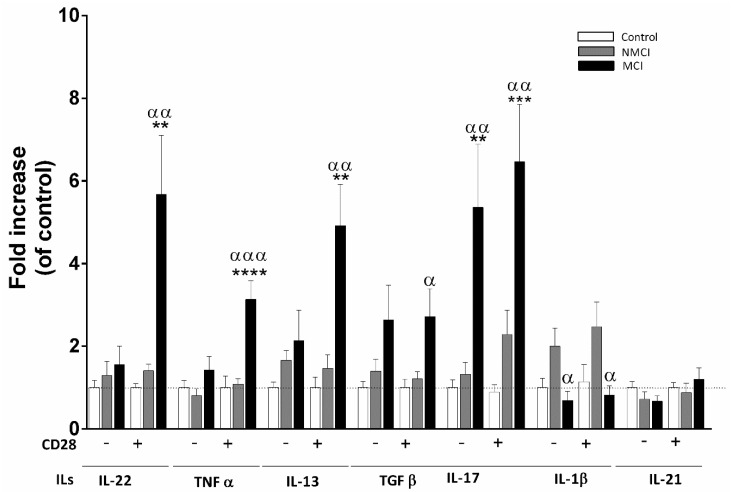
Analysis of cytokines released by CD4^+^ T cell cultures of in the absence (−) or presence (+) of added anti-CD28. Values are expressed as the fold increase of the cytokine levels over the levels in the controls, which were considered as 1. Values are the mean ± SEM of the following groups: control, n = 12; NMCI, n = 13; MCI, n = 12. Values differing significantly from controls are indicated by asterisks (*). Values significantly different in patients with MCI compared to NMCI patients are indicated by (^α^): ^α^ *p* < 0.05; **/^αα^ *p* < 0.01; ***/^ααα^ *p* < 0.001; **** *p* < 0.0001). MCI, mild cognitive impairment.

CD4^+^ T-lymphocyte subsets were best characterized by incubating isolated CD4^+^ T lymphocytes in the absence or presence of added anti-CD28 and measuring the cytokines released into the culture medium. Generally, no significant changes were observed in the cytokines released from the NMCI patients compared to the controls, while a marked increase of several cytokines was found for the patients with MCI (Figure 5). The TNFα, IL-22, IL-17, and IL-13 release increased significantly after activation with anti-CD28 in cultures of cells from the MCI patients compared to CD4^+^ cells from the controls or the NMCI patients. It is noteworthy that, in the absence of added anti-CD28, the release of IL-17 was already strongly increased in CD4^+^ T cultures from the MCI patients but not in those from the NMCI patients, suggesting an association between increased activation of Th17 lymphocytes and IL-17 production and appearance of MCI (Figure 5). TFG-β was increased in the cultures from the MCI patients in the absence and presence of added anti-CD28, but the increase compared to the concentration in patients without MCI only reached significance after activation (Figure 5). No differences were found for IL-21 and IL-1β in patients with MCI compared with the controls. However, the NMCI patients showed a significant increase in IL-1β release in both basal and activation conditions compared to the patients with MCI (*p* < 0.05) (Figure 5).

### 2.7. Logistic Regression Analyses of Predictors of the Presence of MCI in NAFLD Patients

In this study, we identified several immunological and inflammatory parameters altered in NAFLD patients with MCI. As shown in Table 2, there were significant correlations between most of these parameters and the diagnostic score for mild cognitive impairment.

On univariate analysis, MCI in the NAFLD patients was significantly associated with the plasma levels of IL-13 and the percentage of activated autoreactive cells (CD4^+^CD28^−^ CD69^+^) (Table 2). Multivariate logistic regression analysis, using the presence of MCI as the dependent variable and the parameters that were significant in univariate analyses as independent variables, showed that only IL-13 concentration was significantly associated with MCI in the NAFLD patients (OR: 1.459; 95% confidence interval 1.111–1.916; *p* = 0.007) (Table 2).

The ROC curve analysis of IL-13 for the diagnosis of MCI in the NAFLD patients showed an AUC value of 0.804 (95% confidence interval 0.680–0.928; *p* < 0.0001) (Figure S2). At the cutoff of 6.98 pg/mL, the specificity was 83%, and the sensitivity was 68%. 

## 3. Discussion

**Figure 6:**
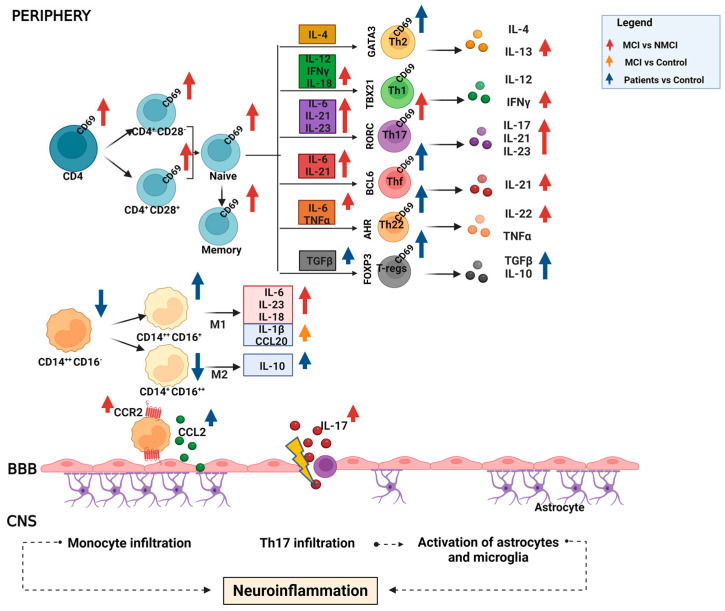
Scheme summarizing MCI- and NAFLD-associated changes in immunophenotype and inflammation. The main alterations associated with MCI are: (1) increased activation of CD4^+^ T lymphocytes, as indicated by increased CD69 expression; (2) increased activation of Th17 cells and increased plasma levels of IL-17, which could cause an alteration of the blood–brain barrier (BBB) favoring the infiltration into the CNS of peripheral immune cells, including Th17 cells. In the CNS, IL-17 exerts a direct effect on neurons and oligodendrocytes, inducing damage, and also activates astrocytes and microglia, promoting neuroinflammation; (3) increased IL-13, as a compensatory anti-inflammatory response to the increased expression of pro-inflammatory cytokines; (4) increased CCL2 and CCR2, which may promote monocytes infiltration into the CNS. Upward arrows: increase; downward arrows: decrease.

Our data demonstrate that the appearance of MCI in patients with NAFLD is associated with specific changes in the immune system and inflammation, which differ from those present in patients without MCI, as summarized in Figure 6.

The NAFLD patients with MCI showed an increase in CD69 in CD4^+^ T cells. Within the CD4^+^ T cell subsets, the activation of Th17 cells stands out, which also shows spontaneous and selective activation in CD4^+^ T cell cultures obtained from patients with MCI, as indicate the increased levels of IL-17. These results suggest an association between increased activation of Th17 lymphocytes and IL-17 production and the appearance of MCI in NAFLD patients.

CD69 is an early activation marker of T lymphocyte due to its rapid appearance on the surface of activated cells after the engagement of the T cell receptor (TCR) by an antigenic peptide [30]. Multiple studies reported CD69 expression on infiltrating lymphocytes at inflammatory sites in several chronic human inflammatory conditions [31,32,33]. CD69 also regulates several specific T cell subset functions, specifically, Treg cell differentiation as well as IFN-γ, IL-17, and IL-22 secretion [33]. Increased CD69 expression in lymphocytes from MCI patients is therefore a marker of several signaling pathways, potentially regulating their activated phenotype and differentiation, resulting in immune dysfunction and altered expression of several cytokines. 

The appearance of MCI in patients with NAFLD is associated with higher plasma levels of several pro-inflammatory and anti-inflammatory cytokines and with an abnormal differentiation of CD4^+^ T-helper subsets. In particular, Th17 lymphocytes are activated by a distinct set of pro-inflammatory cytokines, among which IL-6, IL-21, and IL-23 are essential for developing Th17, while IL-17 is produced by Th17 cells [34]. The key role of Th17 cells in the pathogenic mechanism of several inflammatory disorders and brain diseases, including multiple sclerosis, ischemic brain injury, and Alzheimer’s disease, is well documented [35,36,37,38]. However, the pathogenic effect exercised by Th17 cells and their characteristic cytokine IL-17 on the CNS remains incompletely understood. The patients with MCI showed high plasma levels of the cytokines IL-6, IL-22, IL-23 and, especially, IL-17 compared with the patients without MCI. In addition, CD4^+^ T cells isolated from the patients with MCI released a greater amount of IL-17 in vitro, and Th17 cells were more activated in the patients with MCI than in the other study groups. An indirect role of IL-17 in promoting early and subclinical atherosclerosis in obese patients has been reported [39], which could be a trigger of MCI in NAFLD patients. A recent study demonstrated the mechanisms of action of Th17 cells and IL-17 in the CNS [35]. The IL-17 receptor (IL-17R) is present in different cell types in the CNS, and circulating IL-17 causes the breakdown of the blood–brain barrier (BBB) by altering the tight junctions and the expression of cell adhesion molecules in endothelial cells, favoring the entry of Th17 cells, neutrophils, and other peripheral immune cells into the CNS [38].

We showed an infiltration of peripheral T lymphocytes CD4^+^, CD4^+^CD28^−^, and Th17 and Tfh in the cerebellar meninges of patients with steatohepatitis associated with the activation of microglia and astrocytes and the loss of Purkinje and granular neurons [27,28]. The high plasma level of IL-17 indicated Th17 activation in the circulation and could cause the disruption of the BBB, allowing the infiltration of Th17 cells into the CNS and thus contributing to cognitive and motor alterations.

The plasma levels of IL-13 increased only in the patients with MCI and were predictive of MCI in the NAFLD patients. In addition, the IL-13 release in vitro was significantly increased from CD4^+^ T cells isolated from the MCI patients compared to those from patients without cognitive impairment. Studies have linked the IL-13 levels with the progression of NAFLD to NASH, given the higher IL-13 levels in NASH patients than in NAFLD patients. This increase is due to the direct induction of IL-13 via the expression of profibrotic genes (such as collagens or connective tissue growth factor) in hepatic stellate cells [40]. IL-13 has been reported to induce tissue fibrosis by stimulating and activating TGFβ [41]. IL-13 is a Th2 cytokine in the immune system thought to play an important role in the development of allergies, although it has also been ascribed anti-inflammatory roles, inhibiting different pro-inflammatory mediators such as IL-1β and TNF-α [42]. It also antagonizes the actions of IFN-γ, another pro-inflammatory cytokine [43]. Previous studies showed that IL-13 altered the activation state of microglia/macrophages towards the protective M2-like phenotype polarized in neurodegenerative disorders [44,45]. Increased IL-13 appears to be a compensatory anti-inflammatory response to the increased expression of several pro-inflammatory cytokines in patients with MCI, such as IL17 [46]. 

MCI patients had elevated levels of CCL2, also referred to as monocyte chemoattractant protein 1 (MCP-1), and its receptor, the chemokine receptor CC2 (CCR2), compared to patients without MCI. CCR2 is expressed on monocytes and T lymphocytes and is the most potent chemokine in regulating the migration and infiltration of monocytes/macrophages [47]. The CCR2 receptor plays an important role in certain neurodegenerative diseases such as Alzheimer’s disease and multiple sclerosis, where it mediates aberrant cell migration [48,49]. In Alzheimer’s disease, CCL2 is primarily expressed by microglia and macrophages, which are involved in β-amyloid removal, myelin degradation, and neuronal loss [50]. Patients with MCI and mild Alzheimer’s disease exhibited elevated serum CCL2 and cerebrospinal fluid levels, which likely represents an early event in the pathogenesis of Alzheimer’s disease, far preceding the clinical onset of the disease [51].

Likewise, in models of hepatic inflammation, it was found that microglia are activated and generate CCL2, promoting the infiltration of monocytes into the CNS [52].

The elevated levels of CCL2 and its CCR2 receptor in NAFLD patients with MCI could favor the infiltration of monocytes into the CNS. In addition, increased CCL20 levels in MCI patients, although not statistically significant, may also contribute to T-lymphocyte infiltration, as reported in previous studies [27,28]. This infiltration could be intensified in patients with MCI due to the alterations in their immune system affecting the integrity of the BBB, caused especially by the action of IL-17 [38].

As summarized in Figure 6, the results of this study highlight changes in the immune system of NAFLD patients with MCI and suggest possible mechanisms involved in the development of this impairment. The main alterations associated with MCI were: (1) increased activation of CD4^+^ T lymphocytes, as indicated by increased CD69 expression; (2) enhanced activation of Th17 cells and increased plasma levels of IL-17, which may cause a BBB rupture, favoring the entry of peripheral immune cells, including Th17 cells, into the CNS. In the CNS, IL-17 has a direct effect on neurons and oligodendrocytes, inducing damage, and activates astrocytes and microglia, promoting neuroinflammation; (3) increased IL-13, as a compensatory anti-inflammatory response to the increased expression of pro-inflammatory cytokines; (4) increased CCL2 and CCR2, which may promote the infiltration of monocytes in the CNS.

Patients with cognitive impairment experience a significant impact on their quality of life, despite the apparent absence of clinical signs or symptoms. Early detection could improve and restore cognitive functions and health-related quality of life in NAFLD patients who have MCI. In this regard, our findings from the analysis of MCI pathophysiology in NAFLD patients could provide a springboard from which to design new treatments to reverse MCI and its associated neurological alterations.

## 4. Materials and Methods

### 4.1. Patients and Controls

A total of 71 patients with NAFLD were consecutively recruited from the outpatient clinics of the Clínico and Arnau de Vilanova hospitals in Valencia, Spain. NAFLD diagnosis was evaluated from clinical, biochemical, and ultrasonographic data. Exclusion criteria were psychiatric or neurological diseases; recent (<6 weeks) use of drugs that affect the cognitive function; recent (<6 months) alcohol intake; recent (<6 weeks) antibiotic use or gastrointestinal bleeding; other liver disease or hepatocellular carcinoma. A total of 61 healthy volunteers were included in the study once liver disease was ruled out by clinical, analytical, and serological tests. The subjects included in the study did not have fever or any clinical or biological signs of recent infection. Blood ammonia was measured immediately after blood collection with the PocketChemBA System Ammonia II Test Kit (Arkay, Inc., Kyoto, Japan) on the same day as the psychometric tests were performed. All participants were included in the study after signing a written informed consent. The study was conducted according to the guidelines of the Declaration of Helsinki and approved by the Scientific and Research Ethics Committees of the Hospital Clínico Universitario and Arnau de Vilanova Hospital of Valencia, Valencia, Spain (approval code: 2018/123; approval date: 25 July 2019).

### 4.2. NAFLD Patient Classification into NAFL or NASH

Liver biopsies were available from 48 of the 71 NAFLD patients, who were classified as NAFL or NASH using the NAFLD activity score (NAS), as described by Kleiner et al. [53]. Biopsy extraction was not clinically justified in the remaining 23 patients, who were classified according to the FibroScan-AST (FAST) score [54].

### 4.3. MCI Diagnosis 

The neurological functions of patients and controls were evaluated by psychometric tests, such as Oral SDMT, d2, Stroop test, digit span, and visual–motor or bimanual coordination [6]. MCI was diagnosed using a new sensitive score developed for patients with NAFL [6] and was graded according to the Adams and Foley criteria [55]. The cut-off between normal and pathological results in the test battery was set at −5 points. 

### 4.4. Characterization of Leukocyte Populations by Flow Cytometry

For the general study of leukocyte, naïve, memory, and autoreactive T-helper lymphocyte populations, venous blood samples were placed in BD Vacutainer^®^ tubes with EDTA (Becton, Dickinson and Company, Franklin Lakes, NJ, USA). Then, 50 µL of whole blood was incubated with a mixture of monoclonal antibodies specific for the different leukocyte subpopulations (see below) and with 2 mL of BD FACS Lysing Solution (Becton, Dickinson and Company, Franklin Lakes, NJ, USA). The samples were incubated in the dark for 10 min at room temperature, after which, 50 µL of Flow Count (Beckman Coulter, Miami, FL, USA) was added to quantify the number of cells per microliter. T-helper lymphocyte (CD4^+^) subpopulations were studied in isolated peripheral blood mononuclear cells (PBMC). Dead cells were excluded from analysis by adding 100 µL of Zombie Violet (Zombie Fixable Viability Kit, Biolegend, San Diego, CA, USA) to the samples as a viability marker, followed by incubation for 30 min in the dark at room temperature. PBMCs (300,000 cells) were incubated with a mixture of monoclonal antibodies specific to the different CD4^+^ cell subpopulations (see below) and with 2 mL of VersaLyse Lysing Solution (Beckman Coulter). The samples were incubated in the dark for 10 min at room temperature. Next, 100 µL of Flow Count (Beckman Coulter, Miami, FL, USA) was added to quantify the number of cells per microliter. The analysis was performed with a Gallios flow cytometer (Beckman Coulter, Miami, FL, USA), and the Kaluza software package was used to analyze the flow cytometry data.

### 4.5. Monoclonal Antibodies

Different cell populations were labeled with antibodies against CD45 (total leukocytes), CD14 and CD16 (monocytes), CD3 (T lymphocytes), CD4 (T helper lymphocytes), CD69 (activated lymphocytes). Several populations of interest were identified among T-helper lymphocytes (CD4^+^): autoreactive T helper lymphocytes (CD28^−^), naïve and memory T lymphocytes (CD45RA and CD45RO), Thf (CXCR5), Th1 (CXCR5^−^/CXCR3^+^), Th2 (CXCR3^−^/CCR4^+^/CCR10^+^/CCR6^−^), Th22 (CXCR3^−^/CCR4^+^/CCR10^+^), Th17 (CCR4^+^/CCR6^+^), and Tregs (CD25^+^/FoxP3^+^). The monoclonal antibodies used are shown in Table S1.

### 4.6. Determination of Plasma Cytokine Levels

The blood samples were centrifuged for 10 min at 1500× *g* to obtain plasma samples which were kept at −80 °C for subsequent cytokine analysis. The levels of IL-8, IL-18, IL-23, IL-4, TGF-β, IL-22, IL-1β, IL-10, IL-15, CCL20, CCL2, CCL5, CX3CL1, and BDNF were measured by DuoSet^®^ ELISA Kits (R&D Systems, Minneapolis, MN, USA). The IL-13 levels were measured by the Human IL-13 ELISA kit (Invitrogen, Thermo Fisher, Waltham, MA, USA). High-sensitivity kits were required to evaluate IL-6 (Human IL-6 Quantikine HS ELISA Kit, R&D Systems, Minneapolis, MN, USA). The concentrations of TNF-α, IL-12p70, IL-17A, and IFN-γ were measured with cytokine 6-plex Panel 1 (TNF-α, IL-12p70, IL-17A, IL-10, IL-6, and IFN-γ) (IFN-γ, IL-6, IL-10, IL-12p70, IL-17A, TNF-a) (Quanterix Corp., Billerica, MA, USA) using SIMOA SR-X equipment (Quanterix Corp., Billerica, MA, USA). Additional measurements for IL-17A were performed with the IL-17A 2.0 Advantage Assay using SIMOA™ HD-X equipment (Quanterix Corp., Billerica, MA, USA).

### 4.7. Analysis of Transcription Factors by Quantitative PCR

RNA was extracted from PBMC with an RNAspin mini-RNA isolation kit according to the manufacturer’s instructions (GE Healthcare, Buckinghamshire, UK). The RNA was retro-transcribed to cDNA with the High-Capacity cDNA Reverse Transcription Kit (Applied Biosystems, Foster City, CA, USA). For real-time PCR (40 cycles), Gene Expression Master Mix and the following Taqman^®^ assays labeled with the FAM dye were used: TBX21 (Hs00203436_m1), GATA3 (Hs00231122_m1), BCL6 (Hs00153368_m1), RORC (Hs01076122_m1), FOXP3 (Hs01085834_m1) AHR (Hs00907314_m1), CCR2 (Hs00704702_s1), TLR2 (Hs01872448_s1), and TLR4 (Hs00152939_m1). The ΔΔCt method was used to determine the expression of the targets, using HPRT1 (Hs02800695_m1) as a housekeeping gene. All reagents were from Applied Biosystems (Foster City, CA, USA).

### 4.8. Analysis of Cytokines Released by CD4^+^ Lymphocyte Cultures

CD4^+^ T lymphocytes were isolated from frozen PBMC using the EasySep™ Human CD4^+^ T Cell Isolation Kit (Stem Cell Technologies, Vancouver, BC, Canada). CD4^+^ cells were resuspended at a concentration of 1 × 10^6^ cells/mL in X-VIVO 20 serum-free medium (Lonza Group Ltd., Basel, Switzerland) supplemented with 1% penicillin/streptomycin. CD4^+^ cells (500,000 cells/well) were incubated in 48-well plates previously coated with an anti-CD3 antibody (Becton Dickinson, Franklin Lakes, NJ, USA) with or without a CD28 antibody (Becton Dickinson, Franklin Lakes, NJ, USA), which induces cell activation. After 6 h of incubation at 37 °C with 5% CO_2_, the supernatant and cell pellets were collected for further analysis. The cytokines IL-17, IL-22, TNF-α, TFG-β, and IL-1β were measured in the culture supernatant by DuoSet^®^ ELISA Kits (R&D Systems, Minneapolis, MN, USA). For the IL-13 measurement, a human IL-13 ELISA kit was used (Invitrogen, Thermo Fisher, USA). IL-21 interleukin was measured in the cell pellet by western blot.

### 4.9. Statistical Analysis

Continuous variables are reported as mean and standard error of the mean (SEM) and comparisons performed using the Student’s *t*-test or one-way analysis of variance (ANOVA) followed by post-hoc Tukey’s multiple comparison test. Categorical data were analyzed by the Chi-squared test. Bivariate correlations were evaluated using the Spearman’s Rho correlation test. Univariate and multivariate logistic regressions were performed using MCI as the dependent variable. Potential explanatory variables used in univariate analysis were those showing significant (*p* < 0.05) differences between NMCI patients and MCI patients. Multivariate logistic regression analysis was performed including, as independent variables, those parameters that were significant in univariate analysis. Receiver operating characteristic (ROC) curves were obtained to determine the sensitivity and specificity of the predictor variables found. The results were analyzed with GraphPad PRISM vs. 8 and SPSS vs. 28.0 (SPSS Inc., Chicago, IL, USA). The probability level accepted for significance was *p* < 0.05. 

## Figures and Tables

**Table 1:** Characteristics of the patients and controls included in this study.

Parameter	Controls	NAFLD Patients without MCI	NAFLD Patients with MCI	*p* Value
Number of subjects [n (% of total patients)]	61	51 (71)	20 (29)	
Age ^a^	58 ± 1.0	58 ± 1.1	59 ± 2.4	0.966
Sex [n (%)]				0.314
Male	34 (53)	26 (51)	8 (40)	
Female	30 (47)	25 (49)	12 (60)	
Education (years of schooling) ^a^	14 ± 0.5	14 ± 0.6	12 ± 1.0	0.181
Comorbidity [n (%)]				
Diabetes mellitus		18 (35)	12 (60)	0.060
Dyslipidemia		30 (59)	12 (60)	0.928
Arterial Hypertension		25 (49)	13 (65)	0.228
Metabolic Syndrome		21 (41)	12 (60)	0.156
Laboratory Parameters ^a^				
AST (U/L)	25.8 ± 1.2	37.8 ± 3.0 **	36.5 ± 3.6 *	0.0012
ALT (U/L)	25.7 ± 1.7	46.5 ± 3.6 ****	45.4 ± 5.1 **	<0.0001
Albumin (g/dL)	4.4 ± 0.04	4.4 ± 0.04	4.3 ± 0.09	0.205
Bilirubin (mg/dL)	0.5 ± 0.03	0.8 ± 0.07	0.6 ± 0.12	0.067
Creatinine (mg/dL)	0.8 ± 0.02	0.8 ± 0.03	0.8 ± 0.07	0.845
Platelets (×10^9^/L)	242.3 ± 9.0	227.0 ± 10.3	292.1 ± 22.8 *^/bb^	0.005
INR	1.01 ± 0.00	1.02 ± 0.01	1.2 ± 0.19	0.099
Ammonia (µM)	9.9 ± 0.5	15.1 ± 1.3 **	18.2 ± 3.1 ***	<0.0001
BMI (Body Mass Index) ^a^	29 ± 1.8	32 ± 0.7	32.4 ± 1.5	0.415
MCI Score ^a^	−0.8 ± 0.3	−1.0 ± 0.3	−7.2 ± 0.5 ****^/bbbb^	<0.0001
Diagnosis [n (%)]				
NAFL		27 (53)	12 (60)	0.593
NASH		24 (47)	8 (40)	

^a^ Values are expressed as mean ± standard error of the mean (SEM). Between-group comparisons were performed using ANOVA followed by post-hoc Tukey’s test for continuous data and Chi-square (𝜒^2^) test for categorical data. Abbreviations: ALT, alanine aminotransferase; AST, aspartate aminotransferase; BMI, body mass index; INR, international normalized ratio; MCI, mild cognitive impairment; NAFL, non-alcoholic fatty liver; NAFLD, non-alcoholic fatty liver disease; NASH, non-alcoholic steatohepatitis. Values significantly different from controls are indicated by asterisks (*). Values significantly different in patients with vs. patients without MCI are indicated by (b): * *p* < 0.05; **/^bb^ *p* < 0.01; *** *p* < 0.001; ****/^bbbb^ *p* < 0.0001.

**Table 2:** Correlations between immunological and inflammatory parameters and MCI score and univariate and multivariate logistic regression analyses to predict MCI in NAFLD patients.

**Correlations with MCI Score**		
**Parameter**	**Correlation Coefficient**	***p* Value**
IL-10	−0.342	0.001
IL-6	−0.318	0.007
IL-13	−0.417	<0.0001
IL-17A	−0.323	0.001
CCL2	−0.244	0.035
RORC	0.419	0.012
CCR2	−0.410	0.011
CD4^+^ (CD69^+^)	−0.254	0.039
CD4^+^ naïve (CD69^+^)	−0.246	0.046
CD4^+^CD28^−^ (CD69^+^)	−0.309	0.012
CD4^+^CD28^+^ (CD69^+^)	−0.290	0.022
**Univariate Logistic Regression Analyses**		
**Independent Variables**	**OR (95% CI)**	***p***
IL-6	1.06 (0.87–1.29)	0.560
**IL-13**	**1.37 (1.12–1.67)**	**0.002**
IL-17A	110.6 (0.65–18,952)	0.07
IL18	1.002 (0.999–1.005)	0.280
IL21	1.026 (0.993–1.060)	0.128
IL22	1.000 (1.000–1.001)	0.439
IL23	1.024 (1.000–1.048)	0.051
INFγ	3.95 (0.75–20.7)	0.105
BDNF	1.000 (1.000–1.001)	0.077
CD4^+^ memory	1.037 (0.986–1.090)	0.156
CD4^+^ (CD69^+^)	1.172 (0.997–1.377)	0.055
CD4^+^ naïve (CD69^+^)	1.116 (0.917–1.359)	0.272
CD4^+^ memory (CD69^+^)	1.118 (0.987–1.266)	0.080
**CD4^+^CD28^−^ (CD69^+^)**	**1.084 (1.002–1.173)**	**0.044**
CD4^+^CD28^+^ (CD69^+^)	1.142 (0.987–1.322)	0.075
Th17 (CD69^+^)	0.960 (0.848–1.088)	0.525
**Multivariate Logistic Regression Analyses**		
**Predictor Variables**	**OR (95% CI)**	***p***
IL-13	1.459 (1.111–1.916)	0.007

Correlation coefficient and *p* value for Spearman’s Rho correlations are shown. On both uni- and multivariate analyses, the dependent variable was the presence of mild cognitive impairment (MCI). On multivariate analysis, the independent variables were those parameters that were significant (*p* < 0.05) on univariate analysis (in bold). BDNF, brain-derived neurotrophic factor; CI, confidence interval; IL, interleukin; OR, odds ratio.

## Data Availability

The data are contained within the article and the Supplementary Materials.

## Supplementary Materials

The supporting information can be downloaded at: https://www.mdpi.com/article/10.3390/ijms241210407/s1.

## References

[1] Serfaty L., Lemoine M. (2008). Definition and natural history of metabolic steatosis: Clinical aspects of NAFLD, NASH and cirrhosis. Diabetes Metab..

[2] Marchesini G., Bugianesi E., Forlani G., Cerrelli F., Lenzi M., Manini R., Natale S., Vanni E., Villanova N., Melchionda N. (2003). Nonalcoholic fatty liver, steatohepatitis, and the metabolic syndrome. Hepatology.

[3] Younossi Z.M., Koenig A.B., Abdelatif D., Fazel Y., Henry L., Wymer M. (2016). Global epidemiology of nonalcoholic fatty liver disease-Meta-analytic assessment of prevalence; incidence; and outcomes. Hepatology.

[4] Celikbilek A., Celikbilek M., Bozkurt G. (2018). Cognitive assessment of patients with nonalcoholic fatty liver disease. Eur. J. Gastroenterol. Hepatol..

[5] Kjærgaard K., Mikkelsen A.C.D., Wernberg C.W., Grønkjær L.L., Eriksen P.L., Damholdt M.F., Mookerjee R.P., Vilstrup H., Lauridsen M.M., Thomsen K.L. (2021). Cognitive Dysfunction in Non-Alcoholic Fatty Liver Disease-Current Knowledge; Mechanisms and Perspectives. J. Clin. Med..

[6] Giménez-Garzó C., Fiorillo A., Ballester-Ferré M.P., Gallego J.J., Casanova-Ferrer F., Urios A., Benlloch S., Martí-Aguado D., San-Miguel T., Tosca J. (2021). A new score unveils a high prevalence of mild cognitive impairment in patients with nonalcoholic fatty liver disease. J. Clin. Med..

[7] Montoliu C., Piedrafita B., Serra M.A., del Olmo J.A., Urios A., Rodrigo J.M., Felipo V. (2009). IL-6 and IL-18 in blood may discriminate cirrhotic patients with and without minimal hepatic encephalopathy. J. Clin. Gastroenterol..

[8] Díaz-Gerevini G.T., Repossi G., Dain A., Tarres M.C., Das U.N., Eynard A.R. (2014). Cognitive and motor perturbations in elderly with longstanding diabetes mellitus. Nutrition.

[9] Shin S.Y., Julian L., Katz P. (2013). The relationship between cognitive function and physical function in rheumatoid arthritis. J. Rheumatol..

[10] Cabrera-Pastor A., Llansola M., Montoliu C., Malaguarnera M., Balzano T., Taoro-Gonzalez L., García-García R., Mangas-Losada A., Izquierdo-Altarejos P., Arenas Y.M. (2019). Peripheral inflammation induces neuroinflammation that alters neurotransmission and cognitive and motor function in hepatic encephalopathy: Underlying mechanisms and therapeutic implications. Acta Physiol..

[11] Mangas-Losada A., García-García R., Leone P., Ballester M.P., Cabrera-Pastor A., Urios A., Gallego J.J., Martínez-Pretel J.J., Giménez-Garzó C., Revert F. (2019). Selective improvement by rifaximin of changes in the immunophenotype in patients who improve minimal hepatic encephalopathy. J. Transl. Med..

[12] Magaki S., Yellon S.M., Mueller C., Kirsch W.M. (2008). Immunophenotypes in the circulation of patients with mild cognitive impairment. J. Psychiatr. Res..

[13] Saresella M., Calabrese E., Marventano I., Piancone F., Gatti A., Alberoni M., Nemni R., Clerici M. (2011). Increased activity of Th-17 and Th-9 lymphocytes and a skewing of the post-thymic differentiation pathway are seen in Alzheimer’s disease. Brain Behav. Immun..

[14] Mangas-Losada A., García-García R., Urios A., Escudero-García D., Tosca J., Giner-Durán R., Serra M.A., Montoliu C., Felipo V. (2017). Minimal hepatic encephalopathy is associated with expansion and activation of CD4^+^CD28^−^; Th22 and Tfh and B lymphocytes. Sci. Rep..

[15] Petranovic D., Pilcic G., Valkovic T., Sotosek Tokmadzic V., Laskarin G. (2014). Perforin- and granulysin-mediated cytotoxicity and interleukin 15 play roles in neurocognitive impairment in patients with acute lymphoblastic leukaemia. Med. Hypotheses.

[16] Zindler E., Zipp F. (2010). Neuronal injury in chronic CNS inflammation. Best Pract. Res. Clin. Anaesthesiol..

[17] Tosello-Trampont A.C., Landes S.G., Nguyen V., Novobrantseva T.I., Hahn Y.S. (2012). Kuppfer cells trigger nonalcoholic steatohepatitis development in diet-induced mouse model through tumor necrosis factor-production. J. Biol. Chem..

[18] Lefere S., Tacke F. (2019). Macrophages in obesity and non-alcoholic fatty liver disease: Crosstalk with metabolism. JHEP Rep..

[19] Van Herck M.A., Weyler J., Kwanten W.J., Dirinck E.L., De Winter B.Y., Francque S.M., Vonghia L. (2019). The Differential Roles of T Cells in Non-alcoholic Fatty Liver Disease and Obesity. Front. Immunol..

[20] Chackelevicius C.M., Gambaro S.E., Tiribelli C., Rosso N. (2016). Th17 involvement in nonalcoholic fatty liver disease progression to non-alcoholic steatohepatitis. World J. Gastroenterol..

[21] Shawcross D.L., Davies N.A., Williams R., Jalan R. (2004). Systemic inflammatory response exacerbates the neuropsychological effects of induced hyperammonemia in cirrhosis. J. Hepatol..

[22] Felipo V., Urios A., Montesinos E., Molina I., Garcia-Torres M.L., Civera M., Olmo J.A., Ortega J., Martinez-Valls J., Serra M.A. (2012). Contribution of hyperammonemia and inflammatory factors to cognitive impairment in minimal hepatic encephalopathy. Metab. Brain Dis..

[23] Civera M., Urios A., Garcia-Torres M.L., Real J.T., Ortega J., Martinez-Valls J., Cassinello N., del Olmo J.A., Rodrigo J.M., Montoliu C. (2010). Relationship between insulin resistance, inflammation and liver cell apoptosis in patients with severe obesity. Diabetes Metab. Res. Rev..

[24] Felipo V., Urios A., García-Torres M.L., El Mlili N., del Olmo J.A., Civera M., Ortega J., Ferrandez A., Martínez-Valls J., Cassinello N. (2013). Alterations in adipocytokines and cGMP homeostasis in morbid obesity patients reverse after bariatric surgery. Obesity.

[25] Higarza S.G., Arboleya S., Gueimonde M., Gomez-Lazaro E., Arias J.L., Arias N. (2019). Neurobehavioral dysfunction in non-alcoholic steatohepatitis is associated with hyperammonemia; gut dysbiosis; and metabolic and functional brain regional deficits. PLoS ONE.

[26] Weinstein G., Zelber-Sagi S., Preis S.R., Beiser A.S., DeCarli C., Speliotes E.K., Satizabal C.L., Vasan R.S., Seshadri S. (2018). Association of Nonalcoholic Fatty Liver Disease With Lower Brain Volume in Healthy Middle-aged Adults in the Framingham Study. JAMA Neurol..

[27] Balzano T., Forteza J., Molina P., Giner J., Monzó A., Sancho-Jiménez J., Urios A., Montoliu C., Felipo V. (2018). The Cerebellum of Patients with Steatohepatitis Shows Lymphocyte Infiltration; Microglial Activation and Loss of Purkinje and Granular Neurons. Sci. Rep..

[28] Balzano T., Forteza J., Borreda I., Molina P., Giner J., Leone P., Urios A., Montoliu C., Felipo V. (2018). Histological Features of Cerebellar Neuropathology in Patients With Alcoholic and Nonalcoholic Steatohepatitis. J. Neuropathol. Exp. Neurol..

[29] Kim D.G., Krenz A., Toussaint L.E., Maurer K.J., Robinson S.A., Yan A., Torres L., Bynoe M.S. (2016). Non-alcoholic fatty liver disease induces signs of Alzheimer’s disease (AD) in wild-type mice and accelerates pathological signs of AD in an AD model. J. Neuroinflamm..

[30] Vilanova M., Tavares D., Ferreira P., Oliveira L., Nóbrega A., Appelberg R., Arala-Chaves M. (1996). Role of monocytes in the up-regulation of the early activation marker CD69 on B and T murine lymphocytes induced by microbial mitogens. Scand. J. Immunol..

[31] Afeltra A., Galeazzi M., Ferri G.M., Amoroso A., De Pità O., Porzio F., Bonomo L. (1993). Expression of CD69 antigen on synovial fluid T cells in patients with rheumatoid arthritis and other chronic synovitis. Ann. Rheum. Dis..

[32] Crispin J.C., Martinez A., de Pablo P., Velasquillo C., Alcocer-Varela J. (1998). Participation of the CD69 antigen in the T-cell activation process of patients with systemic lupus erythematosus. Scand. J. Immunol..

[33] Cibrián D., Sánchez-Madrid F. (2017). CD69: From activation marker to metabolic gatekeeper. Eur. J. Immunol..

[34] Ouyang W., Kolls J.K., Zheng Y. (2008). The biological functions of T helper 17 cell effector cytokines in inflammation. Immunity.

[35] Kebir H., Kreymborg K., Ifergan I., Dodelet-Devillers A., Cayrol R., Bernard M., Giuliani F., Arbour N., Becher B., Prat A. (2007). Human TH17 lymphocytes promote blood-brain barrier disruption and central nervous system inflammation. Nat. Med..

[36] Oberstein T.J., Taha L., Spitzer P., Hellstern J., Herrmann M., Kornhuber J., Maler J.M. (2018). Imbalance of circulating Th17 and regulatory T cells in Alzheimer’s disease: A case control study. Front. Immunol..

[37] Dolati S., Ahmadi M., Khalili M., Taheraghdam A.A., Siahmansouri H., Babaloo Z., Aghebati-Maleki L., Jadidi-Niaragh F., Younesi V., Yousefi M. (2018). Peripheral Th17/Treg imbalance in elderly patients with ischemic stroke. Neurol. Sci..

[38] Cipollini V., Anrather J., Orzi F., Iadecola C. (2019). Th17 and Cognitive Impairment: Possible Mechanisms of Action. Front. Neuroanat..

[39] Tarantino G., Costantini S., Finelli C., Capone F., Guerriero E., La Sala N., Gioia S., Castello G. (2014). Is serum Interleukin-17 associated with early atherosclerosis in obese patients?. J. Transl. Med..

[40] Liu Y., Munker S., Müllenbach R., Weng H.L. (2012). IL-13 Signaling in Liver Fibrogenesis. Front. Immunol..

[41] Lee C.G., Homer R.J., Zhu Z., Lanone S., Wang X., Koteliansky V., Shipley J.M., Gotwals P., Noble P., Chen Q. (2001). Interleukin-13 induces tissue fibrosis by selectively stimulating and activating transforming growth factor beta(1). J. Exp. Med..

[42] Miossec P., van den Berg W. (1997). Th1/Th2 cytokine balance in arthritis. Arthritis Rheum..

[43] Kolosowska N., Keuters M.H., Wojciechowski S., Keksa-Goldsteine V., Laine M., Malm T., Goldsteins G., Koistinaho J., Dhungana H. (2019). Peripheral administration of IL -13 induces anti-inflammatory microglial/macrophage responses and provides neuroprotection in ischemic stroke. Neurotherapeutics.

[44] Hamzei Taj S., Le Blon D., Hoornaert C., Daans J., Quarta A., Praet J., Van der Linden A., Ponsaerts P., Hoehn M. (2018). Targeted intracerebral delivery of the anti-inflammatory cytokine IL13 promotes alternative activation of both microglia and macrophages after stroke. J. Neuroinflamm..

[45] Gordon S. (2003). Alternative activation of macrophages. Nat. Rev. Immunol..

[46] Newcomb D.C., Boswell M.G., Zhou W., Huckabee M.M., Goleniewska K., Sevin C.M., Hershey G.K., Kolls J.K., Peebles R.S. (2011). Human TH17 cells express a functional IL-13 receptor and IL-13 attenuates IL-17A production. J. Allergy Clin. Immunol..

[47] Sozzani S., Zhou D., Locati M., Rieppi M., Proost P., Magazin M., Vita N., van Damme J., Mantovani A. (1994). Receptors and transduction pathways for monocyte chemotactic protein-2 and monocyte chemotactic protein-3. Similarities and differences with MCP-1. J. Immunol..

[48] Reale M., Iarlori C., Feliciani C., Gambi D. (2008). Peripheral chemokine receptors; their ligands; cytokines and Alzheimer’s disease. J. Alzheimers Dis..

[49] Fantuzzi L., Tagliamonte M., Gauzzi M.C., Lopalco L. (2019). Dual CCR5/CCR2 targeting: Opportunities for the cure of complex disorders. Cell Mol. Life Sci..

[50] Britschgi M., Wyss-Coray T. (2007). Systemic and acquired immune responses in Alzheimer’s disease. Int. Rev. Neurobiol..

[51] Galimberti D., Fenoglio C., Lovati C., Venturelli E., Guidi I., Corrà B., Scalabrini D., Clerici F., Mariani C., Bresolin N. (2006). Serum MCP-1 levels are increased in mild cognitive impairment and mild Alzheimer’s disease. Neurobiol. Aging.

[52] D’Mello C., Le T., Swain M.G. (2009). Cerebral microglia recruit monocytes into the brain in response to tumor necrosis factor signaling during peripheral organ inflammation. J. Neurosci..

[53] Kleiner D.E., Brunt E.M., Van Natta M., Behling C., Contos M.J., Cummings O.W., Ferrell L.D., Liu Y.C., Torbenson M.S., Unalp-Arida A. (2005). Non alcoholic Steatohepatitis Clinical Research Network. Design and validation of a histological scoring system for nonalcoholic fatty liver disease. Hepatology.

[54] Newsome P.N., Sasso M., Deeks J.J., Paredes A., Boursier J., Chan W.K., Yilmaz Y., Czernichow S., Zheng M.H., Wong V.W. (2020). FibroScan-AST (FAST) score for the non-invasive identification of patients with non-alcoholic steatohepatitis with significant activity and fibrosis: A prospective derivation and global validation study. Lancet Gastroenterol. Hepatol..

[55] Adams R.D., Foley J.M. (1953). The neurological disorders associated with liver disease. Proc. Assoc. Res. Nerv. Ment. Dis..

