# Supplementary material for: Mild Cognitive Impairment Is Associated with Enhanced Activation of Th17 Lymphocytes in Non-Alcoholic Fatty Liver Disease
Source: Int J Mol Sci. 2023 Jun 20;24(12):10407. doi: 10.3390/ijms241210407 (PMC10298852; doi:10.3390/ijms241210407)
Supplement: Supplementary file 1 [file ijms-24-10407-s001.zip › ijms-2443418-supplementary.pdf]

SUPPLEMENTARY INFORMATION

Mild cognitive impairment is associated with immunological and inflammatory alterations in non-alcoholic fatty liver disease

Alessandra Fiorillo, Juan-José Gallego, Franc Casanova-Ferrer, Carla Giménez-Garzó, Amparo Urios, Maria-Pilar Ballester, Lucia Durbán, Maria-Pilar Rios, Javier Megías, Teresa San Miguel, Elena Kosenko, Desamparados Escudero-García, Salvador Benlloch, Vicente Felipo, Carmina Montoliu*

Contents:

Table S1: Monoclonal antibodies used

Table S2. Test performance of controls and patients

Table S3. Characteristics of patients grouped by liver damage

Table S4. Monocyte and $CD4^+$ lymphocyte populations in controls and patients grouped by liver damage (NAFL and NASH)

Table S5. Plasma cytokine levels in controls and patients

Table S6. Plasma cytokine levels of NAFL and NASH patients.

Figure S1. Monocyte populations in peripheral blood from NAFLD patients and controls.

Figure S2. Receiver operating characteristic (ROC) curves for sensitivity and specificity of IL-13, and activated autoreactive cells (%$CD69^+$) in the diagnosis of mild cognitive impairment in NAFLD patients.

Table S1. Monoclonal antibodies used

Antibodies	Fluorochrome	Clone	Company
**CD45**	KO (Krome Orange)	J33	Beckman Coulter
**CD14**	PB (Pacific Blue)	M5E2	Biolegend
**CD16**	APC (Allophycocyanin) -Alexa Fluor* 750	3G8	Beckman Coulter
**CD3**	APC (Allophycocyanin)	UCHT1	Biolegend
**CD4**	ECD (Electron Coupled Dye)	SFCI12T4	Beckman Coulter
**CD45RA**	PERCP/Cy5.5 (Peridinin Chlorophyll Protein/Cyanin5.5)	D11	Biolegend
**CD45RO**	APC/Cy7 (Allophycocyanin/Cyanin7)	HI100	Biolegend
**CD28**	PB (Pacific Blue)	UCHL1	Biolegend
**CXCR5**	Alexa Fluor® 700	J252D4	Biolegend
**CXCR3**	Vio® Bright FITC	REA232	Miltenyi Biotec
**CCR4**	PE-Vio® 770	REA279	Miltenyi Biotec
**CCR6**	Brilliant Violet 510	G034E3	Biolegend
**CCR10**	PE	REA326	Miltenyi Biotec
**CD25**	Brilliant Violet 510	PC61	Biolegend
**FoxP3**	Alexa Fluor® 488	259D/C7	BD Pharmingen
**CD69**	Alexa Fluor® 488	FN50	Biolegend

Table S2. Test performance of controls and patients

TEST /parameter	CONTROLS (n=31)	NAFLD patients without MCI (n=51)	NAFLD patients with MCI (n=20)	Global *p Value*
**Coordination tests**				
**Bimanual (min)**	1.93 ± 0.05	2.03 ± 0.04	2.42 ± 0.09****/bbbb	**<0.0001**
**Visuo-motor (min)**	2.27 ± 0.05	2.26 ± 0.05	2.72 ± 0.11***/bbbb	**<0.0001**
**d2 Test**				
**TR Values**	435 ± 10	420 ± 12	272 ± 20****/bbbb	**<0.0001**
**TA Values**	173 ± 4	159 ± 4	101 ± 8****/bbbb	**<0.0001**
**O Values**	9 ± 1	14 ± 1	15 ± 4	0.0942
**C Values**	1 ± 0.3	1 ± 0.2	5 ± 1.2****/bbb	**<0.0001**
**O+C Values**	10 ± 1	14 ± 1	19 ± 4*	**0.0155**
**TOT Values**	425 ± 10	4402 ± 10	253 ± 19****/bbbb	**<0.0001**
**CON Values**	169 ± 5	157 ± 5	92 ± 7****/bbbb	**<0.0001**
**Stroop Test**				
**Congruent Task (Number of words)**	115 ± 3	111 ± 2	90 ± 3****/bbbb	**<0.0001**
**Neutral Task (Number of colours)**	86 ± 3	84 ± 2	69 ± 3***/bbb	**0.0002**
**Incongruent Task (Number of items)**	49 ± 2	46 ± 1	38 ± 2***/bb	**0.0004**
**Oral SDMT test**				
**Total items**	55 ± 1	53 ± 1	38 ± 2****/bbbb	**<0.0001**
**Correct pairings**	54 ± 1	52 ± 1	36 ± 2****/bbbb	**<0.0001**
**Errors**	0.5 ± 0.1	0.8 ± 0.2	1.7 ± 0.5*	**0.0404**
**DIGIT SPAN Test**				
**Digits forward (right answers)**	9 ± 0.4	9 ± 0.3	8 ± 0.5*	**0.0358**
**Digits backward (right answers)**	7 ± 0.4	6 ± 0.3*	4 ± 0.4***/b	**0.0002**
**Number-letter sequencing Test (right answers)**	10 ± 0.4	9 ± 0.5	7 ± 0.6***/b	**0.0004**

Values are expressed as mean ± SEM (standard error of mean). Results were analyzed with one-way ANOVA followed by post-hoc Tukey's multiple comparisons test. MCI, mild cognitive impairment;

NAFL: non-alcoholic fatty liver; NASH; Oral SDMT, Symbol digit modalities test (oral version). D2 test parameters: TR, Total number of characters processed; TOT, Total correctly processed; CON, Concentration performance; RA, Total right answers; O, errors of omission; C, errors of commission; O+C, Total errors. Values significantly different from controls are indicated by asterisks*. Values significantly different in patients with MCI compared to patients without MCI are indicated by b. ($^{*/b}$ $p < 0.05$; ^{bb} $p < 0.01$; $^{***/bbb}$ $p < 0.001$; $^{****/bbbb}$ $p < 0.0001$).

Table S3. Characteristics of patients grouped by liver damage

Parameter	NAFL patients	NASH patients	p value
**Number of subjects** [n (%)]	39 (55)	32 (45)	
**Age**[a]	58±1.4	59±1.4	0.628
**Sex** [n (%)]			0.115
- **Male**	22 (56)	12 (38)	
- **Female**	17 (44)	20 (62)	
**Education**[a] (years of schooling)	15±0.7	12±0.7	**0.010**
**Comorbidity** [n (%)]			
- **Diabetes mellitus**	14 (36)	16 (50)	0.235
- **Dyslipidemia**	24 (62)	18 (56)	0.654
- **Arterial Hypertension**	18 (46)	20 (63)	0.172
- **Metabolic Syndrome**	15 (39)	18 (56)	0.138
**Laboratory Parameters**[a]			
- **AST (U/mL)**	33.3±2.6	39.6±3.1	0.120
- **ALT (U/mL)**	42.1±3.7	49.9±4.5	0.185
- **Albumin (g/dL)**	4.4±0.0	4.3±0.1	**0.045**
- **Bilirubin (mg/dL)**	0.7±0.1	0.7±0.1	0.559
- **Creatinine (mg/dL)**	0.8±0.0	0.8 ±0.0	0.183
- **Platelets (x10^9/L)**	249.1±14.9	240.6±14.8	0.687
- **INR**	1.1±0.1	1.01±0.1	0.335
- **Ammonia (µM)**	16.3±2.2	15.2 ±(1.3	0.677
**BMI**[a]	30±0.7	34.2±1.0	<0.0001
**Diagnosis** [n (%)]			
- **Biopsy (NAS)**	19 (49)	29 (91)	
- **Fibroscan (FAST)**	20 (51)	3 (9)	
**Fibroscan Parameters**[a]			
- **LSM (kPa)**	6.9±0.5	9.9±1.0	**0.006**
- **CAP (dB/M)**	320.8±10.2	326.4±9.3	0.691
**Fibrosis grade [n (%)]**			**0.023**
- **F0**	6 (30)	3 (11)	
- **F1**	5 (50)	5 (18)	
- **F2**	8 (40)	13 (46)	
- **F3**	1 (5)	7 (25)	
**MCI [n (%)]**	12 (31)	8 (25)	0.593
**MCI Score**[a]	-2.2± 0.7	-3.5±0.05	0.041

[a]Data are shown as mean ± SEM (standard error of mean). Between-group comparisons were analyzed by t-student for continuous data and Chi-Square (χ^2) for categorical data. Abbreviations: AST, aspartate aminotransferase; ALT, alanine aminotransferase; BMI, body mass index; CAP, controlled attenuation parameter; FAST, fibroscan-AST score; INR, international normalized ratio; LSM, liver stiffness measurement; NAFL, non-alcoholic fatty liver; NAS, NAFLD activity score; NASH, non-alcoholic steatohepatitis; MCI, mild cognitive impairment.

Table S4. Monocyte and CD4^{+} lymphocyte populations of controls and patients grouped by liver damage (NAFL and NASH)

	Control	NAFL patients	NASH patients	Global *p value*
**Monocytes**				
**CD14^{++}CD16^{-} (Classical)**	95.2 ± 0.3	93.4 ± 0.5	93.0 ± 0.7*	0.022
**CD14^{+}CD16^{+} (Intermediate)**	2.4 ± 0.2	6.4 ± 0.5****	7.3 ± 0.6****	< 0.0001
**CD14^{+}CD16^{++} (Non-classical)**	0.7 ± 0.1	0.1 ± 0.0****	0.1 ± 0.0****	< 0.0001
**T *helper* lymphocytes**				
**CD4+**	61.9 ± 1.9	62.2 ± 2.6	65.5 ± 2.7	0.569
**CD4+ naïve**	27.5 ± 2.4	36.7 ± 3.3	34.3 ± 3.6	0.063
**CD4^{+} memory**	68.1 ± 2.7	69.5 ± 2.3	73.6 ± 3.6	0.415
**CD4^{+} CD28^{-}**	11.2 ± 1.5	6.0 ± 1.0**	5.5 ± 1.0**	0.002
**CD4^{+} CD28^{+}**	89.0 ± 1.5	95.5 ± 0.9***	94.9 ± 1.3**	0.0004
Activation				
**CD4^{+}**	0.33 ± 0.03	6.04 ± 0.80****	5.15 ± 0.90****	< 0.0001
**CD4^{+} naïve**	0.3 ± 0.1	2.8 ± 0.4****	2.8 ± 0.6****	< 0.0001
**CD4^{+} memory**	0.6 ± 0.1	8.8 ± 1.1****	7.3 ± 1.1****	< 0.0001
**CD4^{+} CD28^{-}**	0.3 ± 0.1	8.1 ± 1.2****	8.4 ± 1.9****	< 0.0001
**CD4^{+} CD28^{+}**	2.5 ± 0.1	6.3 ± 0.8***	5.3 ± 1.0*	0.001

Values are expressed as mean ± SEM (standard error of mean). Results were analyzed with one-way ANOVA followed by post-hoc Tukey's multiple comparisons test. NAFL: non-alcoholic fatty liver; NASH; non-alcoholic steatohepatitis. Values significantly different from controls are indicated by asterisks (*): * $p < 0.05$; ** $p < 0.01$; *** $p < 0.001$; **** $p < 0.0001$). Activation is expressed as the percentage of cells expressing CD69 marker.

Table S5. Plasma cytokine levels in controls and patients

Cytokine concentration (pg/mL*)*	Control	NMCI patients	MCI patients	Global *p value*
**IL-13**	3.6 ± 0.5	4.0 ± 0.4	11.1 ± 2.0****$^{/bbbb}$	<0.0001
**IL-17A**	0.2 ± 0.01	0.2 ± 0.02	0.3 ± 0.003***$^{/bb}$	0.0007
**IFN γ**	0.22 ± 0.02	0.27 ± 0.03	0.62 ± 0.24*$^{/b}$	0.0114
**IL-6**	1.6 ± 0.1	2.8 ± 0.2*	4.1 ± 0.8****$^{/b}$	<0.0001
**IL-18**	144.9 ± 12.1	190.8 ± 11.7	258.7 ± 32.2	0.0002
**IL-10**	3.3 ± 0.2	5.4 ± 0.3****	5.7 ± 0.7***	<0.0001
**IL-22**	200.0 ± 18.8	276.9 ± 27.0	418.5 ± 64.5***$^{/b}$	0.0014
**TGF β**	4759 ± 243.3	8.38 ± 416.0****	8351 ± 832.4****	<0.0001
**IL-8**	7.3 ± 1.3	18.4 ± 1.6****	15.3 ± 2.1*	<0.0001
**IL-15**	4.7 ± 1.0	6.1 ± 0.9	5.2 ± 1.1	0.5753
**IL-23**	11.5 ± 2.9	14.9 ± 2.6	34.2 ± 8.5**$^{/bb}$	0.0026
**IL-12 p70**	0.17 ± 0.00	0.19 ± 0.02	0.14 ± 0.01	0.2364
**TNF α**	5.4 ± 0.2	6.5 ± 0.4	6.5 ± 0.9	0.1186
**IL-4**	65.0 ± 2.3	65.0 ± 2.1	68.4 ± 2.1	0.5108
**IL-21**	8.2 ± 2.3	31.0 ± 4.3*	62.4 ± 19.6***$^{/b}$	0.0006
**IL-1 β**	2.2 ± 0.4	3.7 ± 0.5	4.9 ± 1.0**	0,0104
**CCL5**	7352 ± 830.5	10605 ± 847.6*	11625 ± 1539*	0.0226
**CX3CL1**	489.2 ± 121.9	799.4 ± 116.8	516.6 ± 141.3	0.0154
**CCL20**	8.2 ± 1.6	12.0 ± 1.8	17.9 ± 4.5*	0.1686
**CCL2**	5.1 ± 0.8	11.5 ± 0.8****	12.7 ± 1.5****	0.0369
**BDNF**	1942 ± 159.4	1994 ± 143.5	2624 ± 165.7	<0.0001

Values are expressed as mean ± SEM (standard error of mean). Results were analyzed with one-way ANOVA followed by post-hoc Tukey's multiple comparisons test. MCI, mild cognitive impairment. Values significantly different from controls are indicated by asterisks*. Values significantly different in patients with MCI compared to patients without MCI (NMCI) are indicated by ^{b}. ($^{*/b}$ $p < 0.05$; $^{**/bb}$ $p < 0.01$; *** $p < 0.001$; $^{****/bbbb}$ $p < 0.0001$).

Table S6. Plasma cytokine levels of NAFL and NASH patients

Cytokine concentration (pg/mL	Control	NAFL patients	NASH patients	Global *p value*
**IL-13**	3.6 ± 0.5	6.2 ± 1.0	6.1 ± 1.2	0.166
**IL17A**	0.18 ± 0.02	0.21 ± 0.02	0.20 ± 0.02	0.599
**IFN γ**	0.18 ± 0.2	0.37 ± 0.13	0.39 ± 0.10	0.216
**IL-6**	1.6 ± 0.1	2.9 ± 0.4*	3.6 ± 0.4***	0.0005
**IL-18**	145 ± 12	219 ± 21**	202 ± 14	0.006
**IL-10**	3.3 ± 0.2	5.1 ± 0.3***	6.0 ± 0.6***	<0.0001
**IL-22**	200 ± 19	313 ± 41	353 ± 44*	0.031
**TGF β**	4759 ± 243	8265 ± 464****	7920 ± 648****	<0.0001
**IL-8**	7.3 ± 1.3	18.03 ± 1.8***	16.7 ± 1.9**	<0.0001
**IL-15**	4.7 ± 1.0	5.9 ± 1.3	5.9 ± 1.0	0.665
**IL-23**	11.5 ± 2.9	21.9 ± 4.9	18.2 ± 4.1	0.241
**IL-12p70**	0.17 ± 0.00	0.17 ± 0.02	0.18 ± 0.02	0.983
**TNF alfa**	5.4 ± 0.2	5.8 ± 0.3	7.5 ± 0.8**/b	0.003
**IL-4**	65.0 ± 2.3	67.8 ± 2.1	63.1 ± 2.4	0.389
**IL-21**	8.2 ± 2.3	36.3 ± 9.5*	36.5 ± 5.6*	0.02
**IL-1 beta**	2.2 ± 0.4	4.2 ± 0.7 *	3.6 ± 0.5	0.03
**CCL5**	7352 ± 830.5	11475 ± 1006**	10136 ± 1111	0.012
**CX3CL1**	489 ± 122	547 ± 83	937 ± 173	0.039
**CCL20**	8.2 ± 1.6	14.6 ± 3.0	13.0 ± 2.2	0.131
**CCL2**	5.1 ± 0.8	11.7 ± 1.0****	12.0 ± 0.9****	<0.0001
**BDNF**	1942 ± 159	2399 ± 135	1783 ± 205[b]	0.025

Values are expressed as mean ± SEM (standard error of mean). Results were analyzed with one-way ANOVA followed by post-hoc Tukey's multiple comparisons test. NAFL: non-alcoholic fatty liver; NASH, non-alcoholic steatohepatitis. Values significantly different from controls are indicated by asterisks*. Values significantly different between NASH and NAFL patients are indicated by [b]. (*/b $p < 0.05$; ** $p < 0.01$; *** $p < 0.001$; **** $p < 0.0001$).

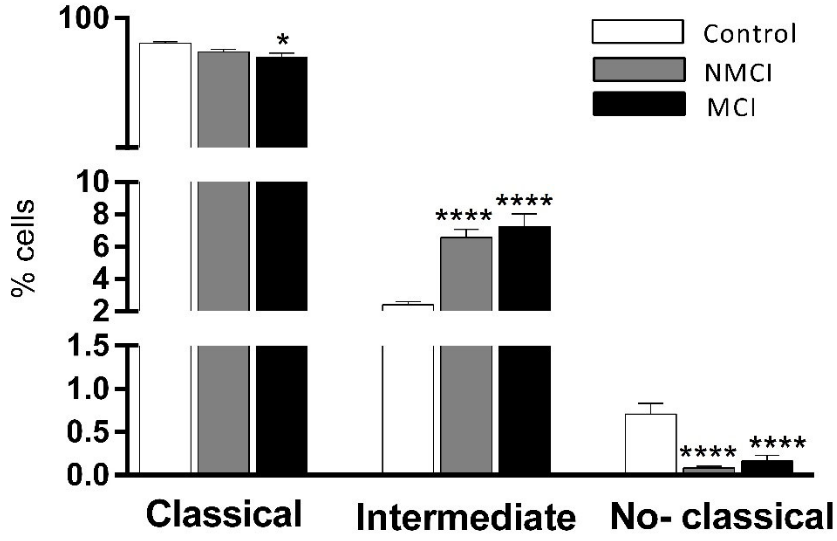

Figure S1. Monocyte populations in peripheral blood from NAFLD patients and controls. Percentage of the three monocyte subsets relative to total monocyte cells: classical ($CD14^{++}CD16^{-}$), intermediate ($CD14^{++}CD16^{+}$) and non-classical ($CD14^{+}CD16^{++}$). Values are mean ± SEM of the following groups: control, n = 15; patients without MCI (NMCI), n = 26; patients with MCI (MCI), n = 12. Values significantly different from controls are indicated by asterisks*. ($^{*}p<0.05$; $^{****}p<0.0001$). MCI, mild cognitive impairment.

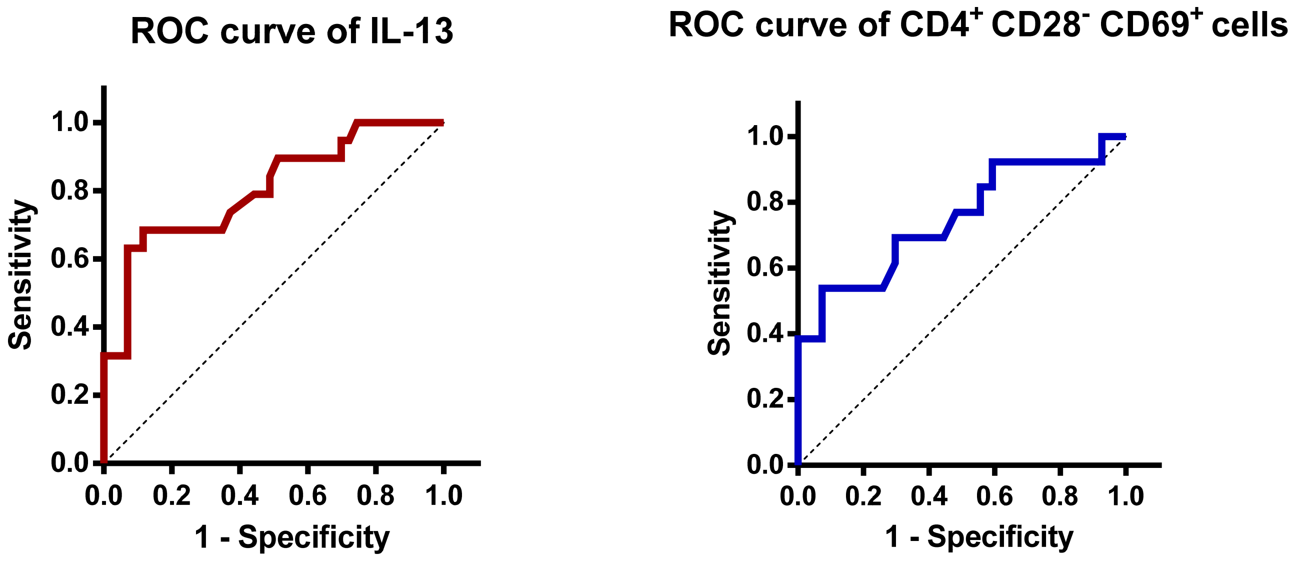

Figure S2. Receiver operating characteristic (ROC) curves for sensitivity and specificity of IL-13 and activated autoreactive cells (%CD69^{+}) in the diagnosis of mild cognitive impairment in NAFLD patients. Area under the curves (AUC) were: IL-13: AUC: 0.804 (95% confidence interval (CI) 0.680-0.928; *P*=0.0001); Activated autoreactive cells: AUC: 0.75 (95% CI 0.575-0.924; *P*=0.01). For IL13, the specificity was 83% and the sensitivity was 68% at a cutoff of 6.98 pg/mL. For activated autoreactive cells, the specificity was 70% and the sensitivity was 69% at a cutoff of 7.86 %.
